# Loneliness in Brazil: a silent threat to Public Health

**DOI:** 10.1590/0102-311XEN229524

**Published:** 2025-07-25

**Authors:** Hisrael Passarelli-Araújo

**Affiliations:** 1 Centro de Desenvolvimento e Planejamento Regional, Universidade Federal de Minas Gerais, Belo Horizonte, Brasil. Centro de Desenvolvimento e Planejamento Regional Universidade Federal de Minas Gerais Belo Horizonte Brazil

**Keywords:** Loneliness, Unified Health System, Population Dynamics, Solidão, Sistema Único de Saúde, Dinâmica Populacional, Soledad, Sistema Único de Salud, Dinámica Poblacional

## Abstract

Loneliness has emerged as a significant Public Health issue, yet its impact in developing countries remains understudied. This study examines the growing concern regarding loneliness in Brazil and how aging, urbanization, and changes in family structure may erode traditional social supports and deepen loneliness across various groups. The study also assesses the challenges of measuring loneliness, given its subjective nature, and critically evaluates how well the Brazilian Unified National Health System (SUS) is equipped to address loneliness via community-based interventions and integrated healthcare strategies. The study suggests that loneliness is intensifying due to demographic changes as the population ages, becomes more urbanized, and experiences shifts in family structure. Although SUS employs community-focused health strategies and extensive care models to combat loneliness, it struggles with challenges like insufficient funding, high patient-to-professional ratios, and a fragmented healthcare system. This study emphasizes that loneliness cannot be resolved medically, such as with vaccine, but with strengthened social connections and community support. We advocate for a comprehensive approach that includes healthcare and cross-sector collaboration with the education, housing, and social services branches to tackle both the symptoms and root causes of loneliness, positioning it as a pressing Public Health priority.

## Resumo

A solidão tem surgido como um tópico relevante para a Saúde Coletiva, mas seu impacto em países em desenvolvimento ainda é pouco estudado. Este estudo examina a crescente preocupação com a solidão no Brasil, e como o envelhecimento, a urbanização e mudanças nas estruturas familiares podem enfraquecer redes de apoio tradicionais e aprofundar a solidão em diversos grupos. O estudo também avalia os desafios de medir a solidão, dada sua natureza subjetiva, e analisa criticamente o quão bem o Sistema Único de Saúde (SUS) está preparado para lidar com a solidão por meio de intervenções comunitárias e estratégias de cuidado integrado. Os resultados sugerem que a solidão está se intensificando devido a mudanças demográficas, à medida que a população envelhece, se urbaniza e passa por transformações nas estruturas familiares. Embora o SUS adote estratégias focadas na saúde da comunidade e modelos de cuidado extensivo para combater a solidão, ele ainda enfrenta desafios como financiamento deficitário, altas proporções de pacientes por profissional e um sistema fragmentado. Este estudo enfatiza que a solidão não pode ser resolvida medicamente, como com uma vacina, mas por meio de conexões sociais fortalecidas e apoio comunitário. Defendemos uma abordagem abrangente que inclua saúde e colaboração intersetorial com educação, habitação e serviços sociais para combater sintomas e causas profundas da solidão, posicionando-a como uma prioridade urgente na Saúde Coletiva.

## Resumen

La soledad se ha convertido en un tema relevante para la Salud Pública, pero su impacto en los países en desarrollo aún está poco estudiado. Este estudio examina la creciente preocupación por la soledad en Brasil y cómo el envejecimiento, la urbanización y los cambios en las estructuras familiares pueden debilitar las redes de apoyo tradicionales y profundizar la soledad en diversos grupos. Además, se evalúan los desafíos de medir la soledad dada su naturaleza subjetiva y se analiza críticamente qué tan bien el Sistema Único de Salud (SUS) está preparado para enfrentar la soledad mediante intervenciones comunitarias y estrategias de atención integrada. Los resultados revelan que la soledad se está intensificando debido a los cambios demográficos a medida que la población envejece, se urbaniza y sufre transformaciones en las estructuras familiares. Aunque el SUS adopta estrategias centradas en la salud comunitaria y en modelos de atención extensiva para combatir la soledad, todavía enfrenta desafíos como la falta de fondos, la alta proporción de pacientes por profesional y un sistema fragmentado. Este estudio enfatiza que la soledad no se puede resolver médicamente con una vacuna, sino mediante conexiones sociales fortalecidas y apoyo comunitario. Abogamos por un enfoque integral que incluya la salud y la colaboración intersectorial con la educación, la vivienda y los servicios sociales para combatir los síntomas y las causas fundamentales de la soledad al situarla como una prioridad urgente en Salud Pública.

## Introduction

Loneliness is an invisible crisis - quiet, pervasive, and hiding in plain sight. Unlike other public health challenges, loneliness leaves no visible scars or dramatic headlines. It affects people at every stage of life, from teenagers glued to screens to seniors living alone ^1^. At its core, loneliness disrupts the fundamental human need for social connection ^2^. As social beings, humans depend on interpersonal relationships for emotional well-being, identity formation, and overall survival ^3^. 

Nearly one in four people worldwide - over a billion individuals - reported feeling lonely, according to a recent Meta-Gallup survey across more than 140 countries in 2022 ^4^. Paradoxically, in an age of technological advancements and instant digital communication, feelings of isolation have become more prevalent ^5^^,^^6^. The pervasive intrusion of work into personal life via emails, messaging applications, and remote working tools has blurred the boundaries between professional and private spheres. The emphasis on productivity and individual success in modern society often comes at the expense of community and relationships, leaving the human need for genuine social interaction unfulfilled.

Loneliness extends beyond physical isolation, which is itself a significant risk factor ^7^. Scientific literature defines loneliness as the distressing feeling that arises when a person’s social network is lacking, either quantitatively or qualitatively ^8^. This definition hinges on perceptions: individuals can lead relatively solitary lives without feeling lonely, or they can have numerous social connections yet still experience loneliness ^9^. 

The recognition of loneliness as a public health concern has grown in recent years. The World Health Organization has already acknowledged its impact on well-being ^10^. In 2017, former U.S. Surgeon General Dr. Vivek Murthy described loneliness as an epidemic, equating its health effects to smoking 15 cigarettes a day ^11^. A year later, the United Kingdom appointed a Minister for Loneliness, making it one of the first countries to formally recognize loneliness as a significant public health concern ^12^^,^^13^. In 2021, Japan followed suit, appointing its own Minister of Loneliness amid rising social isolation exacerbated by the COVID-19 pandemic ^14^. 

Despite the significant health implications of loneliness, it is not yet a formal part of the public health agenda in Brazil. A global survey conducted in 2021 revealed that 50% of Brazilian respondents reported feeling lonely often, always, or sometimes - far above the global average of 33% - despite the relatively high frequency of social interactions in Brazil compared to other nations ^6^^,^^15^^,^^16^^,^^17^. This oversight is especially concerning given the rapid demographic changes and high levels of socioeconomic inequality in the country ^18^. 

Like many developing nations, Brazil faces significant challenges in assessing loneliness due to limited and fragmented data, which hinders efforts to determine its prevalence, identify vulnerable populations, and understand its impact on health outcomes ^19^. Evidence shows that population aging, urbanization, migration, and shifting family structures can amplify loneliness ^20^^,^^21^. These same demographic shifts are rapidly unfolding in Brazil, with serious consequences for both individual health and the public health system in the coming decades. These concerns were first raised in 1987 by Veras et al. ^22^, who warned that social transformations could exacerbate loneliness and poverty among older adults in Brazil - a prediction that remains relevant today. However, loneliness knows no age boundaries; it spans generations, affecting people at different life stages and presenting complex challenges for Public Health.

This essay explores how loneliness poses a silent threat to Public Health, particularly in the context of the demographic shifts that are reshaping Brazilian society. It addresses the challenges in quantifying loneliness and underscores its potential long-term health repercussions. This study also critically evaluates how well the Brazilian Unified National Health System (SUS, aconym in Portuguese) is equipped to address loneliness with community-based interventions and integrated healthcare strategies. By articulating the long-term health risks and the systemic changes needed to mitigate loneliness, we aim to raise awareness among policymakers, healthcare providers, and the public about the urgency of addressing loneliness as a silent threat to Public Health in Brazil.

## How demographic shifts can amplify loneliness

To understand how loneliness poses a threat to Public Health, it is essential to broaden the discussion to include the demographic shifts that Brazil has experienced in recent decades. Population aging, urbanization, migration, and changes in family dynamics have altered how people interact, often amplifying feelings of loneliness. This section discusses each of these factors, examining how they contribute to loneliness and exploring their broader social and health implications. I will look at the vulnerability of the aging population due to reduced social networks, the perceived isolation that can accompany urban living, the disconnection experienced by migrants away from their home communities, and the impact of evolving family structures on traditional support systems.

### Aging population

By 2070, individuals over 60 years of age are projected to represent over 37.8% of the Brazilian population ^23^. This trend is particularly concerning as older adults are more susceptible to loneliness due to several age-related factors ^24^. As they age, many older adults face the loss of peers and spouses, which can reduce their social networks and increase their feelings of loneliness. Retirement compounds this issue by removing daily social interactions that employment provides, leading to increased social disconnection ^25^. Additionally, the decline of physical health and reduced mobility can limit their ability to engage in social activities, further isolating them from community and family interactions ^26^^,^^27^. 

The feminization of aging and the gender paradox in mortality are also a reality in Brazil; despite women tending to have a higher life expectancy and lower mortality rates than men, they report poorer self-rated health than men ^28^. This gender paradox in mortality and health exacerbates the loneliness crisis among older women, as they often face the dual challenges of outliving their social networks and managing chronic health conditions.

The solitude that comes with living alone can lead to loneliness if not accompanied by active social engagement ^29^. This distinction is vital in addressing the social and health implications of aging. Loneliness, particularly when prolonged, is linked to numerous health risks including mental health disorders like depression and anxiety, as well as a physical health decline with conditions like cardiovascular disease and decreased immune function among older adults ^24^.

Despite the *Brazilian Federal Constitution*^30^ of 1988 (article 230) mandating support from family, the community, and the State, the reality on the ground is starkly different. In practice, family care is almost the sole reliable support for many older Brazilians ^31^. Families are generally expected to fully care for their frail members, often without adequate State assistance except in cases of dependent older adults in which the State acts as a punctual partner. Family care falls disproportionately on women and remains the primary support system for most older Brazilians ^32^. This often strains relationships and limits emotional support as women caregivers manage multiple responsibilities and confront their own loneliness, particularly as they outlive male counterparts.

In response, Brazil has established facilities like the Day Center (*Centro Dia*), a public unit specifically designed for the specialized care of older adults and people with disabilities who require some degree of caregiving ^33^. Day Center aims to prevent social isolation, abandonment, and the need for institutional care by fostering an environment of social engagement and providing necessary medical supervision and care. However, despite these efforts, the effectiveness of such services varies widely, and many families continue to face challenges in meeting the comprehensive needs of their older members. Family-provided care falls significantly short of the required needs ^34^, highlighting the critical demand for more structured support systems as the population of older adults grows.

### Urbanization

Urbanization is another key demographic factor that appears to intensify loneliness, as urban environments - characterized by population density, social fragmentation, and individualistic lifestyles - are strongly linked to heightened experiences of loneliness ^35^^,^^36^. In highly urbanized regions, the emphasis on individualism and competitive social interactions often weakens traditional social bonds, increasing social isolation ^35^. Older adults, whether left behind in rural areas or relocated to urban settings, may struggle to form cohesive social networks due to differences in social structures, population density, and cultural values ^37^. The pressures of urban life, including noise, pollution, and overcrowding, further contribute to mental health challenges, including loneliness ^36^. 

Urbanization in Brazil is far from uniform. Large cities like São Paulo and Rio de Janeiro generally attract younger, economically active populations, while smaller municipalities tend to have higher proportions of older adults ^38^. In 2022, census data showed that municipalities with up to 5,000 inhabitants had the highest aging index, with 76.2 older adults for every 100 children aged 0 to 14 ^38^. This demographic imbalance can be attributed to the migration of young people to larger cities, reduced birth rates in smaller towns due to the departure of people of reproductive age, and the return migration of older individuals after retirement ^39^. As a result, many older adults in smaller cities may face greater isolation, weakened social networks, and limited family support.

In urban settings, the pace and lifestyle can lead to less frequent family gatherings and weaker family ties ^40^. Younger family members often prioritize career advancement and the immediate needs of their nuclear families, which may lead to reduced interactions with extended family and increase feelings of isolation among older relatives. Evidence suggests that urban environments, characterized by anonymity and high residential mobility, can weaken social cohesion and limit opportunities for meaningful social interactions ^41^.

While loneliness is often associated with older adults, it also affects other age groups in urban environments. Urban areas often provide greater access to digital technologies, which can paradoxically increase feelings of isolation among teenagers ^42^. Overreliance on virtual interactions can diminish the quality of real-world relationships and contribute to feelings of loneliness and social anxiety ^43^. Adults in urban areas might live in high-rise apartments or gated communities that offer less opportunity for casual, everyday interactions that build relationships ^44^. Furthermore, long commutes in traffic-congested cities can reduce the time available for family interactions and community involvement ^45^.

Although smaller cities offer a more tranquil lifestyle, they are not immune to loneliness. Limited community activities and social opportunities can isolate older adults, teens, and young adults ^46^. For many, adjusting to life changes - such as transitioning to a slower pace, navigating unfamiliar environments, or losing familiar social circles - can be as isolating as life in a large city.

Studies show that urbanization, coupled with rising inequality, disproportionately affects older populations in Brazil, who may experience increased loneliness as family structures change and social networks become fragmented in urban settings ^22^^,^^47^^,^^48^. Poor urban planning exacerbates this issue, as many Brazilian cities lack cohesive community spaces, increasing social isolation among various groups, including adolescents and older adults ^49^^,^^50^. 

### Migration

Migration, whether internal or international, can disrupt social ties, leading to loneliness as individuals leave established communities in search of better opportunities or refuge ^51^. While moving to a new city or country can bring significant career or educational advancements, it frequently comes at the cost of severed social ties and reduced social support. 

Internal migrants in Brazil are frequently move from rural areas or small cities to larger urban centers, driven primarily by economic opportunities and better access to services ^52^^,^^53^. However, migrants might find themselves in unfamiliar environments where forming new friendships and social networks can be challenging ^54^. The initial excitement can give way to feelings of isolation and disconnection from family and familiar surroundings, making loneliness a frequent part of the adaptation process.

Return migration poses additional challenges. Communities may have evolved, former friends may have moved away, and new generations may no longer share the same cultural touchpoints. Such changes leave many feeling like outsiders in places they once called home, especially if they lack close familial ties. For some, this disconnection deepens into “existential loneliness”, which is characterized by feelings of isolation, alienation, emptiness, and abandonment ^55^. Older returnees are particularly vulnerable, reflecting on life choices while confronting their mortality in culturally altered environments with limited social support ^56^. 

For international migrants, the challenges are compounded by cultural and language barriers ^51^. Brazil has experienced a significant influx of migrants and refugees primarily from Venezuela and Haiti, driven by severe political and economic crises in those countries, with Venezuelans constituting the largest group of immigrants in Brazil due to the ongoing Venezuelan refugee crisis ^57^. These migrants often face additional barriers, including language differences, cultural dissonance, and limited access to social services ^58^. The struggle to integrate into Brazilian society and establish meaningful connections can exacerbate their sense of isolation ^57^.

Access to healthcare is another critical issue. Many international migrants lack health insurance and are unfamiliar with the healthcare system in Brazil, which can lead to neglected health needs and increased risk of mental health conditions like anxiety and depression ^59^. Internal migrants also face limited access to mental health services, especially in peripheral urban areas ^60^. Lack of access can leave them without adequate support to manage the psychological stress associated with relocation, increasing their vulnerability to loneliness and related mental health conditions. 

A further complication is the high residential mobility among internal migrants, driven by the search for affordable housing, better job opportunities, or, in extreme cases, survival, such as displacement due to natural disasters ^61^. Studies have shown that frequent moves disrupt the formation of stable social networks, leaving those who move multiple times socially isolated and less likely to establish meaningful relationships with neighbors or community members ^62^. 

Ultimately, whether internal or international, migration highlights the importance of integration and social inclusion. Barriers such as employment instability, illegal immigration status, and cultural differences hinder migrants from fully participating in society, leaving them excluded and more susceptible to loneliness ^63^.

### Family structure changes

Family structures in Brazil have also undergone significant changes in recent decades. Traditionally, Brazilian families were large and multigenerational, with close relationships between parents, children, and extended relatives ^64^^,^^65^. Family was often the cornerstone of social life, with multiple generations living together or maintaining frequent contact. However, demographic shifts such as declining fertility, rising divorce rates, and delayed marriage have led to smaller family sizes and more fragmented family units, reducing the availability of familial support ^66^. 

The Brazilian fertility rate fell from 2.32 children per woman in 2000 to 1.57 in 2023, following global trends toward smaller families ^23^^,^^67^^,^^68^. More Brazilians are also marrying later or choosing to remain single, driven by changing societal values and economic priorities that favor career and personal growth over early marriage ^69^^,^^70^. Between 1980 and 2019, the median age at first marriage rose from 25 to 31 for men and from 22 to 28 for women ^71^^,^^72^. Civil Registry data shows that registered civil marriages declined by 2.7% between 2018 and 2019, reflecting a broader trend of declining marriage rates ^71^. This delay or avoidance of marriage can reduce access to the traditional social support networks that come with family life, increasing the risk of loneliness when individuals face major life challenges alone.

Rising divorce rates also contribute to family fragmentation and weakened social networks. In 2022, Brazil recorded 970,000 marriages and 420,000 divorces - roughly one divorce for every two marriages ^73^. While the emotional toll of divorce is often temporary, losing a partner and breaking intimate connections can leave significant gaps in a person’s support system, amplifying feelings of loneliness during the transition and making recovery more challenging without strong social networks ^74^.

Childlessness in some segments of the population is also becoming more common among Brazilians ^75^, which can have long-term implications for loneliness. Without children, older adults may lack the close familial connections that often provide emotional support and practical help later in life, potentially leading to increased isolation and feelings of loneliness ^76^. In this context, societal changes in family formation patterns can leave individuals more exposed to social isolation, especially as they grow older and their social circles contract.

Household composition has also shifted, with single-person households rising steadily. In 2022, they accounted for over 19% of all households, compared to 12% in 2010 ^77^. While living alone does not inherently cause loneliness, the absence of regular family interaction can heighten the risk, particularly for those without strong external support networks.

Additionally, non-traditional family arrangements, such as cohabiting couples without children and single-parent households, are increasingly prevalent ^78^. While these family structures are not inherently less supportive, they often lack the broader networks that larger, multigenerational families provide. This shift toward smaller and more nuclear family units may foster greater individualism and weaken communal bonds, increasing the likelihood of loneliness ^79^. As a result, individuals in these types of family arrangements may be more susceptible to loneliness, particularly if they do not have strong external support networks from friends or community members.

## Challenges in measuring loneliness in Brazil

Measuring loneliness is challenging due to the subjective nature of the experience and the various factors that influence how individuals perceive and report loneliness. In Brazil, assessing loneliness is particularly complicated due to limited resources and infrastructure. National and local data on loneliness are scarce, hindering efforts to quantify its prevalence and identify vulnerable groups. However, some promising data sources offer valuable insights. The *Brazilian National Health Survey* (PNS), conducted by the Brazilian Institute of Geography and Statistics (IBGE, acronym in Portuguese) in 2019, provides information on health status, lifestyle, and social support. The *Brazilian Longitudinal Study of Aging* (ELSI-Brasil) includes questions related to loneliness, shedding light on its correlation with health and well-being, particularly among older adults ^80^^,^^81^. However, significant gaps remain. 

A crucial distinction in measuring loneliness is separating it from physical isolation. While often used interchangeably, these terms represent distinct concepts. Social isolation is an objective lack of social contact, whereas loneliness is a subjective emotional experience. Survey questions that focus solely on the number of social interactions risk conflating these concepts, leading to inaccurate conclusions. For example, someone with minimal social interactions may not feel lonely if they are content with their level of connection, while a person with many social contacts may report high loneliness if those relationships lack emotional depth. Thus, measuring loneliness requires evaluating not only the quantity of social interactions but also their quality and the individual’s satisfaction with their social connections.

Social stigma presents another challenge in measuring loneliness ^82^. In many cultures, including in Brazil, loneliness is often perceived as a sign of personal failure or social inadequacy. Individuals may fear being perceived as undesirable or socially unworthy if they admit feeling lonely, leading to underreporting in surveys and interviews. This social desirability bias significantly affects the accuracy of loneliness data ^83^, especially in societies that highly value strong family and community bonds. Consequently, loneliness may be far more widespread in Brazil than current estimates suggest.

Existing tools like the *UCLA Loneliness Scale*^84^ and the *De Jong-Gierveld Loneliness Scale*^85^ are widely recognized for their reliability and validity. These self-reported questionnaires ask individuals to rate statements such as “I feel left out” and “I miss having people around me”. While effective in many contexts, applying these scales in culturally diverse settings presents challenges. Cultural factors influence how loneliness is expressed and perceived, underscoring the need for culturally adapted instruments ^86^^,^^87^.

It is essential to distinguish the specific challenges of measuring loneliness from broader issues in psychological assessments across culturally diverse populations. The limitations discussed - such as cultural influences on self-reporting - are not unique to loneliness but reflect the broader complexities of cross-cultural psychological testing ^88^. Emotional expression, language interpretation, and cultural understandings of well-being vary, affecting the reliability of standardized instruments ^89^. In a diverse country like Brazil, culturally sensitive adaptations are crucial for accurate assessments.

Recognizing these challenges, researchers developed the *Brazilian UCLA Loneliness Scale* (UCLA-BR), a culturally adapted scale that significantly advances the measurement of loneliness in Brazil ^90^^,^^91^. The UCLA-BR uses a continuum-based approach to classify loneliness from minimal to severe with well-defined cutoff points, allowing researchers to move beyond binary classifications ^90^. It has demonstrated strong reliability and validity, confirming its usefulness for both research and clinical applications in Brazil.

Additionally, technological innovations are expanding the capacity to track and analyze loneliness. Smartphone apps and social media data analysis provide real-time insights, offering new ways to explore the relationship between loneliness, physical health, social behavior, and emotional well-being ^6^. 

## Challenges faced by the SUS in addressing loneliness

The SUS is one of the largest public health systems in the world, providing universal healthcare access to over 210 million people. While SUS primarily focuses on physical health needs, its comprehensive approach, including mental health services and community-based programs, offers a framework that could be effectively expanded to tackle the growing problem of loneliness.

A central component of SUS is its emphasis on primary healthcare via the Family Health Strategy (FHS). The FHS is a community-based healthcare model that promotes preventive care and fosters strong relationships between healthcare providers and the communities they serve ^92^. This model is particularly effective in identifying and addressing local health needs, including social determinants that contribute to conditions like loneliness.

Healthcare teams within the FHS typically consist of physicians, nurses, community health workers, and other professionals embedded in the communities they serve. These teams maintain continuous contact with families and individuals, enabling them to identify health risks, provide preventive care, and offer personalized support. Such close interaction positions the FHS to detect signs of loneliness and intervene appropriately.

While SUS has the potential to address loneliness via its community-based and preventive healthcare models, it faces numerous challenges that hinder its effectiveness in combating this issue.

### Underfunding 

Although underfunding is a structural issue affecting SUS as a whole, it has implications for addressing complex conditions like loneliness. Rather than being an isolated barrier, financial constraints limit the ability of the system to expand services, invest in infrastructure, and staff healthcare teams ^93^. Public health expenditure in Brazil has historically fallen below the recommended 5% of the Gross Domestic Product (GDP) ^94^, hovering around 3.9% of the GDP in recent years. This shortfall forces SUS to rely on supplementary contributions from state and municipal budgets just to maintain basic operations ^95^.

One of the most significant impacts of underfunding is the scarcity of primary care units and family health teams, particularly in underserved regions such as the North and Northeast ^96^. This shortage limits opportunities for early intervention, counseling, and social support services - critical components in mitigating loneliness and promoting mental health. Additionally, aging infrastructure and inadequate supply chains further compromise the quality of services, affecting everything from basic equipment availability to specialized care for mental health ^92^.

Mental health services are among the most affected. Brazil allocates only about 2-3% of its total health budget to mental health, well below the recommended 5% for middle-income countries ^97^. Although there has been a shift toward community-based care in line with the Psychiatric Reform ^98^, resources remain insufficient to fully integrate mental health programs into primary care ^97^. In 2020, Brazil had only 3.7 psychiatrists per 100,000 people, significantly lower than the 11.3 per 100,000 average across Organisation for Economic Co-operation and Development (OECD) countries ^99^. This figure pales in comparison to high-income nations like Norway and Switzerland, which have over 20 psychiatrists per 100,000 inhabitants ^100^. 

Historic guidelines suggested that 10 psychiatrists per 100,000 people was the minimum threshold to meet a population’s mental health needs, but current estimates indicate that far higher ratios may be necessary given the growing awareness of mental health challenges and gaps in care ^99^. However, without adequate funding, mental health facilities and community programs that could help address loneliness - such as support groups, counseling services, and therapeutic interventions - remain limited in scope and reach.

One practical consequence of underfunding is the limited ability to evaluate and expand community mental health initiatives aimed at combating loneliness. Programs such as Expanded Family Health Care Centers (NASF, acronym in Portuguese), which could offer psychosocial support to socially isolated individuals, have insufficient data and an evaluation process that often fails to meet national guidelines due to the variability in local implementation ^101^. Additionally, research into how loneliness interacts with chronic conditions such as cardiovascular diseases or depression would help SUS design integrated care models that address both physical and mental health.

### High patient-to-professional ratios

Addressing loneliness within SUS is further complicated by imbalances in patient-to-professional ratios and regional disparities. Brazil has fewer healthcare professionals per capita compared to OECD countries, with 2.3 physicians per 1,000 people versus the OECD average of 3.5 ^102^. This low density ranks Brazil among the lowest within the OECD.

High patient-to-professional ratios leave healthcare workers with limited time for meaningful conversations that could help identify and address loneliness. The subjective nature of loneliness requires empathy and active listening - abilities that are difficult to exercise in overburdened settings. Shortages lead to long waiting times and brief consultations, making it harder to detect and manage complex issues like loneliness ^103^. 

Although urban areas have a higher concentration of healthcare professionals, this does not guarantee better management of loneliness. Large cities often suffer from weaker family and community cohesion, reducing social support despite greater access to health services. In contrast, smaller cities and rural areas tend to maintain stronger social ties but lack adequate healthcare infrastructure and professionals to meet complex health needs, including loneliness ^104^.

The uneven distribution of healthcare professionals worsens these challenges. Urban centers face high demand for specialized care, resulting in long waits and fragmented services, while smaller municipalities experience severe shortages of professionals ^105^. Overburdened primary care teams in these areas cannot provide continuous, holistic care, especially for vulnerable groups like older adults and those with chronic conditions.

Many healthcare professionals also lack training in mental health and communication skills. The educational framework in Brazil, influenced by the Flexnerian model, emphasizes hospital-based clinical training and a biomedical focus over community and primary care ^106^. This approach creates a gap between technical knowledge and the need for a more holistic understanding of health that includes social and psychological dimensions. A study by Rocha et al. ^107^ found that primary care physicians often feel unprepared to address mental health issues due to lack of training and resources. Without proper training, healthcare professionals may not recognize signs of loneliness nor acknowledge its impact on health outcomes.

Cultural stigma surrounding mental health further complicates this issue. Societal attitudes may discourage individuals from expressing feelings of loneliness due to fear of judgment, which can also influence healthcare providers’ perceptions and priorities. This stigma contributes to underreporting and underdiagnosis of loneliness and related mental health issues.

### Fragmented approach to care

A fragmented approach to care within SUS poses a significant challenge in addressing loneliness. Healthcare services often operate in silos, resulting in discontinuity of care and a lack of coordination among different levels of the health system ^108^. This fragmentation hinders the implementation of holistic and integrated care models necessary for managing complex issues like loneliness, which span physical, emotional, and social dimensions.

One clear example is the medicalization of psychological suffering. While Brazil has made strides in integrating mental health into primary care via the Psychosocial Care Network (RAPS, acronym in Portuguese), medication remains the dominant treatment due to limited access to alternative therapies, especially in certain regions. Although psychiatric reform aims to reduce excessive medicalization by promoting psychosocial approaches, progress remains uneven ^109^. 

Many patients and healthcare providers are unaware of RAPS or how to access its services ^110^. This lack of integration between primary care and specialized mental health services leaves patients without comprehensive support. Insufficient coordination also limits the effectiveness of community-based interventions crucial for addressing loneliness. Programs designed to foster social interaction and support are difficult to integrate into SUS due to bureaucratic barriers, funding shortages, and weak collaboration between healthcare providers and community organizations ^92^. 

The fragmented approach further burdens families expected to care for individuals with psychological suffering without adequate support from the healthcare system. Many families lack the necessary resources or knowledge to provide effective care. Healthcare professionals sometimes fail to offer proper guidance, assuming families can manage on their own, which overlooks the critical role the healthcare system should play in supporting both patients and caregivers ^111^.

## Concluding remarks

Loneliness poses a growing threat to Public Health in Brazil, driven by significant demographic shifts such as population aging, urbanization, migration, and changing family structures. Loneliness is not exclusive to older populations: it affects individuals across all life stages, from young adults navigating career pressures in urban settings to internal and international migrants rebuilding social connections in unfamiliar environments. Addressing this issue requires a comprehensive approach that spans beyond healthcare to incorporate social policies aimed at enhancing social integration and community support.

One of the key challenges in addressing loneliness is the difficulty in measuring it accurately. The lack of national and regional data, along with the subjective nature of loneliness, complicate efforts to quantify its prevalence and assess its impact on health outcomes. Current research tools, although useful, need to be expanded to capture the complexity of loneliness across different social and cultural settings. Better data is essential for identifying at-risk populations and designing evidence-based interventions.

SUS is pivotal in addressing loneliness with its community-based health strategies and comprehensive care models. However, structural challenges such as underfunding, high patient-to-professional ratios, and fragmented services have hindered its ability to address the emotional and social dimensions of health comprehensively. Strengthening SUS’s capacity to manage loneliness involves not only better funding and resources but also a holistic approach that integrates health services with social programs to foster community ties and support.

Addressing loneliness in Brazil requires a coordinated, multisectoral response. Collaboration between SUS and other sectors - such as education, housing, and social services - could lead to a more comprehensive approach to tackling loneliness. By fostering cross-sector partnerships, Brazil can develop policies that provide healthcare and address the root causes of loneliness, such as poverty, inadequate housing, and lack of community spaces. This comprehensive approach is essential for addressing the silent threat of loneliness to Public Health in the decades ahead.
